# Prominent tauopathy and intracellular β-amyloid accumulation triggered by genetic deletion of cathepsin D: implications for Alzheimer disease pathogenesis

**DOI:** 10.1186/s13195-024-01443-6

**Published:** 2024-04-04

**Authors:** Heather M. Terron, Sagar J. Parikh, Samer O. Abdul-Hay, Tomoko Sahara, Dongcheul Kang, Dennis W. Dickson, Paul Saftig, Frank M. LaFerla, Shelley Lane, Malcolm A. Leissring

**Affiliations:** 1 Institute for Memory Impairments and Neurological Disorders, University of California, Irvine (UCI MIND), Irvine, CA 92697 USA; 2 Department of Neuroscience, Mayo Clinic Florida, Jacksonville, FL 32224 USA; 3 Institut für Biochemie, Christian-Albrechts-Universität zu Kiel, D-24098 Kiel, Germany; 4 Department of Neurobiology and Behavior, University of California, Irvine, Irvine, CA 92697 USA

**Keywords:** Tauopathy, Neurofibrillary tangles, Amyloid-β protein, Cathepsin D, Alzheimer disease, Lysosomes

## Abstract

**Background:**

Cathepsin D (CatD) is a lysosomal protease that degrades both the amyloid-β protein (Aβ) and the microtubule-associated protein, tau, which accumulate pathognomonically in Alzheimer disease (AD), but few studies have examined the role of CatD in the development of Aβ pathology and tauopathy in vivo.

**Methods:**

CatD knockout (KO) mice were crossed to human amyloid precursor protein (hAPP) transgenic mice, and amyloid burden was quantified by ELISA and immunohistochemistry (IHC). Tauopathy in CatD-KO mice, as initially suggested by Gallyas silver staining, was further characterized by extensive IHC and biochemical analyses. Controls included human tau transgenic mice (JNPL3) and another mouse model of a disease (Krabbe A) characterized by pronounced lysosomal dysfunction. Additional experiments examined the effects of CatD inhibition on tau catabolism in vitro and in cultured neuroblastoma cells with inducible expression of human tau.

**Results:**

Deletion of CatD in hAPP transgenic mice triggers large increases in cerebral Aβ, manifesting as intense, exclusively intracellular aggregates; extracellular Aβ deposition, by contrast, is neither triggered by CatD deletion, nor affected in older, haploinsufficient mice. Unexpectedly, CatD-KO mice were found to develop prominent tauopathy by just ∼ 3 weeks of age, accumulating sarkosyl-insoluble, hyperphosphorylated tau exceeding the pathology present in aged JNPL3 mice. CatD-KO mice exhibit pronounced perinuclear Gallyas silver staining reminiscent of mature neurofibrillary tangles in human AD, together with widespread phospho-tau immunoreactivity. Striking increases in sarkosyl-insoluble phospho-tau (∼ 1250%) are present in CatD-KO mice but notably absent from Krabbe A mice collected at an identical antemortem interval. In vitro and in cultured cells, we show that tau catabolism is slowed by blockade of CatD proteolytic activity, including via competitive inhibition by Aβ42.

**Conclusions:**

Our findings support a major role for CatD in the proteostasis of both Aβ and tau in vivo. To our knowledge, the CatD-KO mouse line is the only model to develop detectable Aβ accumulation and profound tauopathy in the absence of overexpression of hAPP or human tau with disease-associated mutations. Given that tauopathy emerges from disruption of CatD, which can itself be potently inhibited by Aβ42, our findings suggest that impaired CatD activity may represent a key mechanism linking amyloid accumulation and tauopathy in AD.

**Supplementary Information:**

The online version contains supplementary material available at 10.1186/s13195-024-01443-6.

## Background

Alzheimer disease (AD) is a progressive, age-related neurodegenerative disorder characterized by extra- and intracellular accumulation of amyloid β-protein (Aβ), intraneuronal aggregates of hyperphosphorylated forms of the microtubule-associated protein, tau, known as neurofibrillary tangles (NFTs), together with extensive neurodegeneration [1]. A wealth of human molecular genetic evidence indicates that specific perturbations to Aβ metabolism are sufficient to trigger the full spectrum of AD-type pathology [2]. Equally strong evidence from other neurodegenerative diseases, on the other hand, shows that tauopathy represents the necessary and proximal cause of neurodegeneration and concomitant clinical symptoms [3]. Understanding how Aβ accumulation contributes to tauopathy, therefore, constitutes one of the most critical topics in the AD field. The precise mechanisms linking Aβ and tauopathy, however, have remained surprisingly elusive.

Cathepsin D (CatD) is an aspartyl protease implicated in the pathogenesis of AD by several independent lines of evidence. First, a coding mutation in the CatD gene (*CTSD*) that disrupts its trafficking [4] has shown significant genetic association with AD risk in a number of studies [5–8], with a recent meta-analysis calculating a statistically significant odds ratio (OR = 1.20; 95% CI = 1.01–1.42; *P* = 0.038) [9]. Second, loss-of-function mutations in *CTSD* in humans and other mammals trigger multiple neurodegenerative diseases, suggesting that impairments in CatD protein levels or function may represent a common mechanism in neurodegeneration [10–12]. Finally, CatD directly degrades both Aβ [9, 13, 14] and tau [15, 16], which accumulate specifically in AD [2]. Despite these and other compelling lines of evidence, however, only a very limited number of studies have investigated the consequences of manipulating CatD on AD-relevant endpoints in animal models [9, 17–20].

We showed previously that CatD knockout (KO) mice [21] develop profound increases in cerebral Aβ relative to wildtype (WT) controls [9]. Although homozygous null CatD-KO mice die prematurely by ∼ 27 days of age due to anorexia [21], significant elevations in endogenous Aβ were evident as early as 15 days of age, well before overt symptomatology [9]. Notably, cerebral Aβ levels in CatD-KO mice exceed those in KO mice lacking either of two well-established Aβ-degrading proteases, neprilysin or insulin-degrading enzyme—or indeed both proteases simultaneously—making CatD the most powerful known mediator of Aβ catabolism in vivo yet identified [9]. Consistent with the subcellular localization of CatD, endogenous Aβ accumulates exclusively within lysosomes in CatD-KO mice to a degree that is readily detectable by standard immunostaining [9]. Crucially, detailed characterization of the enzymological properties of CatD-mediated degradation of Aβ led to the unexpected finding that the longer, more amyloidogenic Aβ species, Aβ42 (but not Aβ40), constitutes a highly potent, subnanomolar, competitive inhibitor of CatD proteolytic activity [9]. This effect was attributable to the combination of an unusually strong, low-nM affinity (K_M_) and an exceptionally slow turnover number (*k*_cat_) [9]. Interestingly, this remarkable length-dependent competitive inhibition of CatD was also shown to extend to shorter Aβ fragments ending in at position 42 (but not 40), including the ⍺-secretase-derived P3 fragment [9], which is produced ∼ 10-fold more abundantly than Aβ42 [22]. These intriguing findings led us to hypothesize that Aβ42 may exert its pathogenic effect in part via inhibition of CatD, which might in turn trigger downstream pathological sequelae [23].

To investigate this hypothesis, here we conducted an extensive characterization of CatD-KO mice in terms of multiple histopathological and biochemical endpoints relevant to AD. In addition to confirming that genetic deletion of CatD triggers profound intracellular accumulation of human Aβ in mice overexpressing human amyloid precursor protein (hAPP), we report that CatD-KO mice exhibit a variety of histological and biochemical features consistent with robust tauopathy, including Gallyas silver staining strongly resembling mature NFTs in AD brain, widespread phospho-tau immunoreactivity, and prominent increases in sarkosyl-insoluble, hyperphosphorylated tau exceeding the levels present in an aggressive transgenic mouse model of tauopathy—all by ∼ 3 weeks of age. We show further that tau catabolism in vitro and in cultured cells is slowed by proteolytic inhibition of CatD, including by Aβ42. To our knowledge, CatD-KO mice represent the only animal model to develop robust tauopathy in the absence of transgenic overexpression of human tau harboring disease-linked mutations [19]. Our findings further implicate CatD deficiency in the pathogenesis of AD and—critically—suggest a plausible mechanistic link between Aβ42 accumulation and downstream pathological sequelae, particularly tauopathy.

## Materials and methods

### Aim, design and setting

The objective of the present study was to evaluate the role of CatD in Aβ and tau proteostasis in vivo and to more completely characterize its role in catabolism of tau. To that end, homogenized brain extracts from CatD-KO mice crossed to hAPP transgenic mice were analyzed to determine steady-state levels of Aβ40 and Aβ42 at different ages and genotypes. Paraffin-embedded brain tissue from CatD-KO mice was analyzed by immunohistochemistry (IHC) for multiple AD-related markers, particularly focused on phospho-tau and other indicators of tauopathy. Western blotting was performed to assess the levels of total tau and various specific tau species in soluble and sarkosyl-insoluble brain extracts, using Krabbe A mice and tau transgenic mice as negative and positive controls, respectively. The extent to which tau catabolism is regulated by CatD proteolytic activity was assessed using an in vitro assay with recombinant tau and a neuroblastoma cell line with tetracycline-regulatable expression of human tau, which were conducted in the absence or presence of a CatD inhibitor or different Aβ species. Research was conducted in multiple state-of-the-art biomedical laboratories.

### Animals

Mice were bred and housed in AAALAC-accredited facilities in accordance with the National Institutes of Health Guidelines for the Care and Use of Laboratory Animals. Lines with genetic deletion of *CTSD* [21] and *GALC* [24], Tg2576 hAPP transgenic mice [25], and JNPL3 human mutant (P301L) tau (hTau) transgenic mice [26] were maintained as inbred lines, each in a mixed C57Bl/6J, DBA genetic background. For the crosses between the CatD-KO and Tg2576 lines, initial matings were performed between (CatD) HET- mice and hAPP transgene-positive (hAPP+) single-transgenic mice then, after 2 generations of backcrossing with HET- mice, experimental cohorts were generated by crossing hAPP-positive, CatD-HET (HET+) mice to hAPP-negative, CatD-HET (HET-) mice. Genotyping was performed using previously published protocols for the CatD-KO [21], GALC-KO [24], JNPL3 [26] and Tg2576 [25] lines. Male mice were used for all experiments.

### Quantification of cerebral Aβ

Cerebral Aβ was extracted from frozen hemibrains as described [27]. Briefly, soluble Aβ was first extracted with 0.2% diethylamine (DEA) followed by centrifugation at 100,000 x g, then insoluble Aβ in the pellet was extracted using guanidium isothiocyanate. Following neutralization and appropriate dilution as described [27], Aβ42 and Aβ40 were quantified using end-specific sandwich ELISAs (Wako Chemicals, Richmond, VA) [28].

### Immunohistochemistry and thioflavin fluorescence

Brain sections were processed and stained within the Mayo Clinic Florida Immunohistochemistry Core. Briefly, freshly dissected hemibrains were fixed by overnight incubation in 4% paraformaldehyde in 0.01 M phosphate buffer, transferred to phosphate buffered saline, embedded in paraffin, cut into 5-µm sections, and mounted on slides. Following deparaffinization, a subset of brain sections was treated with the following histological stains: hematoxylin and eosin (H&E); Gallyas silver stain, with or without hematoxylin counterstain; or thioflavin S, as described [29]. Another subset of brain sections was immunostained with the following primary antibodies (and dilutions, sources): mouse anti-CatD (1:1000; Santa Cruz Biotechnologies, Santa Cruz, CA; Cat. No. sc-377,299); 33.1.1.1, targeting all Aβ species (1:1000; [28, 30]); rabbit anti-ubiquitin (1:1000; Dako North America, Inc., Carpinteria, CA); mouse anti-GFAP (1:2000; Santa Cruz Biotechnologies, Santa Cruz, CA; Cat. No. sc-33,673); CP13, targeting tau phosphorylated at Ser202 (1:1000 or 1:2000; [31]); PHF-1, targeting tau phosphorylated at Ser396 and Ser404 (1:1000 or 1:2000; [32]); or AT180, targeting tau phosphorylated at Thr213 (1:1000; [33]); then visualized by treatment with 3,3’-diaminobenzidine (DAB) with appropriate horse-radish peroxidase-conjugated secondary antibodies, as described [26]. For semi-quantitative analyses, slides were scanned at 20x resolution using a AxioScan.Z1 slide scanner (Carl Zeiss Microscopy, LLC; White Plains, NY), then the resulting images were imported into QuPath (v0.43), in which DAB precipitate, histological staining intensity, or thioflavin S fluorescence was quantified according to manufacturer’s recommendations [34].

### Western blotting

For routine western blotting, soluble protein was extracted as described [9]. For experiments involving phospho-tau protein, brains were rapidly extracted, bisected, and one hemisphere was immediately frozen on dry ice to preserve phosphorylation and sarkosyl-soluble and -insoluble tau were processed and extracted in the presence of phosphatase inhibitors as described [35]. Protein extracts were run on 8% SDS–PAGE gels (ThermoFisher, Waltham, MA), transferred to nitrocellulose and probed with the following antibodies (and dilutions, sources): mouse anti-CatD (1:1000; Santa Cruz Biotechnologies, Santa Cruz, CA; Cat. No. sc-377,299); TAU-5 (1:1000; Abcam, Boston, MA; Cat. No. Ab80579); PHF-1 (1:1000; [32]); C3 (1:500; MilliporeSigma, Burlington, MA; Cat. No. MAB5430-C); P44 (1:1000; [36]); and GAPDH (1:10000; Bio design Meridian Life Science, Memphis, TN; Cat. No. H86504M). Blots were probed with appropriate HRP-conjugated secondary antibodies then visualized by enhanced chemiluminescence and exposure to X-ray film as described [9].

### Tau catabolism

In vitro tau degradation experiments were conducted using recombinant human tau (rTau; generous gift of L. Petrucelli, Mayo Clinic Florida, Jacksonville, FL) and freshly prepared, monomeric Aβ peptides separated from aggregated species by size-exclusion chromatography and characterized as described [37, 38]. Briefly, rTau (200 nM) dissolved in Assay Buffer (60 mM Na-citrate; 80 mM Na_2_HPO_4_, pH 3.5) was combined with Aβ40 or Aβ42 (1 µM) dissolved in Dilution Buffer (20 mM Tris, pH 8.0 supplemented with 0.1% BSA) or equal volumes of Dilution Buffer alone. Reactions were initiated by addition of purified human CatD (2.5 nM; Enzo Life Sciences, Farmingdale, NY) dissolved in Assay Buffer, then 20-µL aliquots were removed 0, 0.5, 1, 2, and 4 h thereafter, with CatD activity in each aliquot immediately terminated by addition of PepA (1 µM) and incubation on ice. Aliquots were separated by conventional SDS-PAGE on 8% polyacrylamide gels subsequently stained with GelCode Blue Stain Reagent according to manufacturer’s recommendations (ThermoFisher, Waltham, MA). Relative protein levels within scanned images of the gels were quantified using ImageJ (v. 1.53k) according to published guidelines [39].

Cell-based tau degradation experiments were performed using the M1C cell model featuring Tet-regulatable (Tet-Off) expression of the human 4R0N hTau isoform [36]. Briefly, cells were plated in 6-well plates at 10% confluency in DMEM containing GlutaMAX^®^ supplemented with 10% fetal bovine serum, 100 U/mL penicillin, and 100 µg/mL streptomycin (ThermoFisher, Waltham, MA), and hTau expression was allowed to occur for 4 days by withdrawal of Tet. Suppression of hTau expression was then initiated by addition of Tet (2 µg/mL; Sigma-Aldrich, St. Louis, MO) combined with PepA (1 µM; Sigma-Aldrich, St. Louis, MO) or vehicle (DMSO) alone, and cells were harvested 0, 1, 2 or 3 days later. Extracts of protein lysates (30 µg/lane), processed and as described previously [36], were separated by SDS-PAGE on 7.5% polyacrylamide gels, transferred to nitrocellulose, then western blotting with the P44 and GAPDH antibodies was performed as described above. Protein levels normalized to GAPDH loading controls were quantified from scanned X-ray film using ImageJ (v1.53k) according to published guidelines [39].

### Statistical analyses

The statistical significance of quantitative data was evaluated in Prism (v10.0.2; GraphPad Software, San Diego, CA) using the 2-tailed Student’s t-test for between-group comparisons, with an alpha level of 0.05 or lower. In the case of tau half-life comparisons, t-tests were run on the errors and means of the respective rate constants.

## Results

**Fig. 1:**
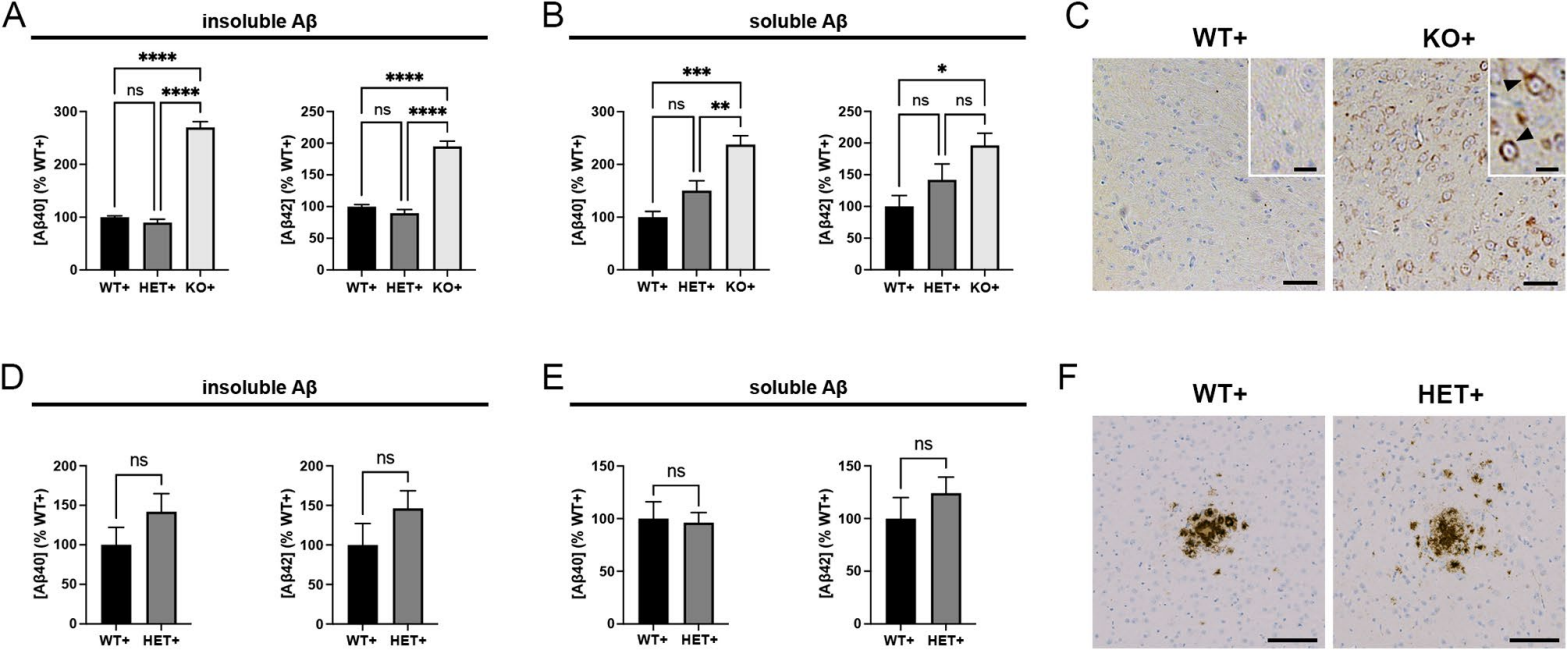
Analysis of Aβ accumulation in CatD-deficient hAPP transgenic mice. (**A**, **B**) Relative levels of insoluble (**A**) and soluble (**B**) Aβ40 (left) and Aβ42 (right) in 3-week-old hAPP transgenic mice with two (WT+), one (HET+), or no (KO+) functional copies of *CTSD* determined by ELISA specific for human Aβ. Note the prominent increases in insoluble Aβ levels in KO+ animals relative to both WT+ and HET+ controls. Data are mean ± SEM of 6 mice per genotype. (**C**) Representative images of pan-Aβ immunoreactivity in WT+ (left) and KO+ (right) brain. Note the prominent accumulation of Aβ in KO+ mice, which occurs exclusively intracellularly (arrowheads), in the absence of extracellular deposits. Scale bars represent 50 μm for the main images and 20 μm for the insets. (**D**, **E**) Relative levels of insoluble (**D**) and soluble (**E**) Aβ40 (left) and Aβ42 (right) in 6- to 10-month-old hAPP transgenic mice with two (WT+) or one (HET+) functional copies of *CTSD* determined by ELISA. No significant differences are evident between genotypes. Data are mean ± SEM of 6–8 mice per group. (**F**) Representative images of Aβ plaques in WT+ (left) and HET+ (right) cortex. Note the lack of differences in plaque size, number, or morphology, as confirmed by morphometric quantification (Sup Fig. 1A-C). *P < 0.05; **P < 0.01; ***P < 0.001; ****P < 0.0001; ns = not significant

To assess the consequences of CatD deletion on accumulation of human Aβ, including potential effects on extracellular deposition, we crossed the CatD-KO line [21] to the Tg2576 line of transgenic mice, which overexpress hAPP harboring the AD-linked Swedish mutation [25]. As was true for endogenous murine Aβ [9], insoluble (guanidinium-extracted) forms of human Aβ40 and Aβ42 were significantly increased in ∼ 3-week-old CatD-KO, hAPP-positive (KO+) mice relative to hAPP-positive mice with two (WT+) or one (HET+) functional copies of *CTSD* (Fig. 1A). Soluble (diethylamine-extracted) forms of both Aβ species were also significantly increased in KO + mice (Fig. 1B).

We previously reported that endogenous, murine Aβ accrues within lysosomes in KO- mouse brain and can be readily detected by conventional Aβ immunostaining [9]. In view of early reports suggesting that anti-Aβ antibodies may interact nonspecifically with lipofuscin [40–42], a lipogenic pigment that accumulates in lysosomes when CatD is deleted [21], we revisited this topic in KO+ mice. In the context of hAPP overexpression, it was evident unambiguously that human Aβ does indeed accumulate in the brains of KO+, but not WT+ or HET+ mice, in the form of intense intracellular Aβ immunoreactivity presenting in a punctate, predominantly perinuclear pattern (Fig. 1C). Of note, we observed no evidence of extracellular Aβ deposition (i.e., amyloid plaques) in KO+ mice (Fig. 1C).

In our prior work, we found that levels of endogenous, murine Aβ—both soluble and insoluble forms—were unchanged in mice lacking one copy of *CTSD* (HET-) relative to wildtype controls (WT-); however, this analysis was limited to mice younger than one month of age [9]. To assess whether *CTSD* haploinsufficiency might affect amyloidogenesis in the context of hAPP overexpression, we quantified Aβ levels in older hAPP-positive mice lacking zero or one functional copy of *CTSD*. Consistent with other reports [9, 18], no significant differences in insoluble (Fig. 1D) or soluble (Fig. 1E) cerebral Aβ levels were observed between 6- to 10-month-old HET+ and WT+ mice. Similarly, we detected no qualitative differences in Aβ plaque morphology (Fig. 1F) or quantitative differences in amyloid plaque number or Aβ-positive area (Sup Fig. 1A-C). ELISA-based quantification of endogenous, murine Aβ levels likewise revealed no differences between 10-month-old (hAPP-negative) HET- and WT- mice, neither insoluble (Sup Fig. 1D) nor soluble (Sup Fig. 1E) forms.

**Fig. 2:**
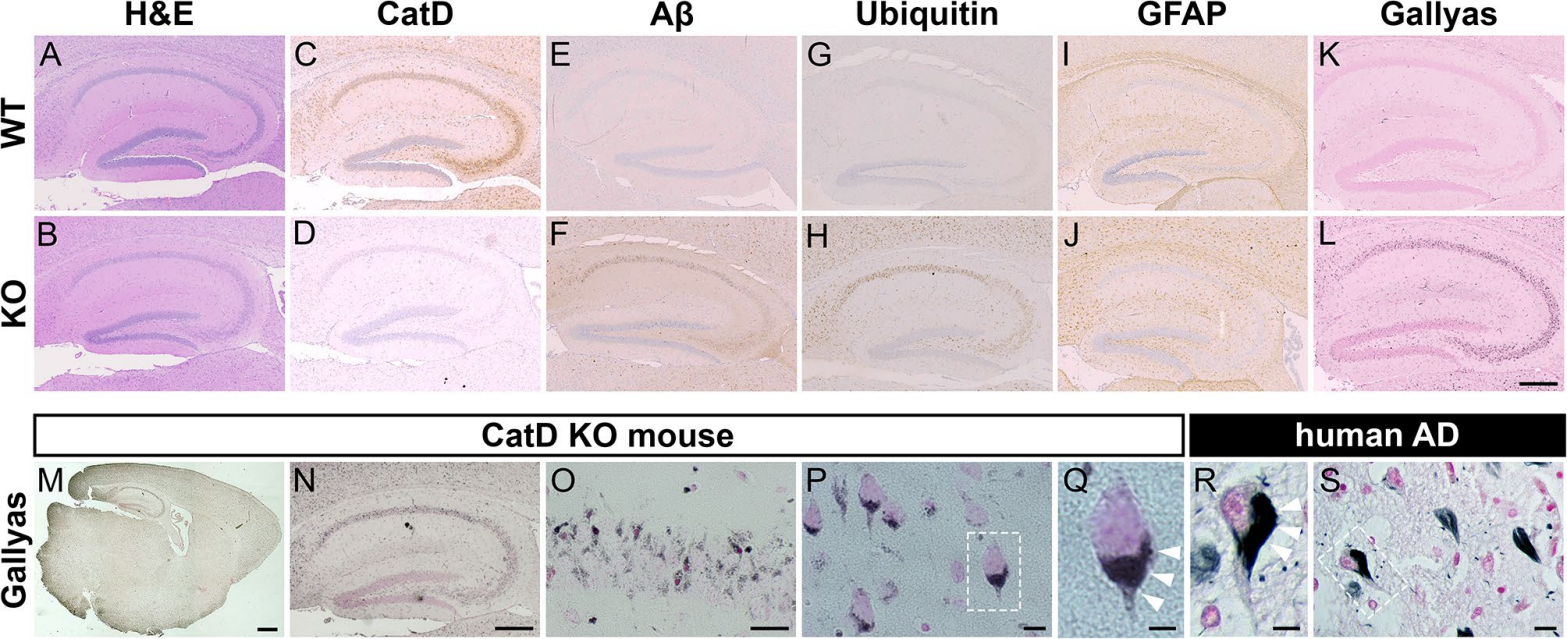
Immunohistochemical characterization of CatD-KO mice. (**A**-**L**) Three-week-old wildtype (WT; top row) and CatD-KO (KO; bottom row) mouse hippocampi stained for hematoxylin and eosin (H&E; **A**, **B**), cathepsin D (CatD; **C**, **D**), pan-Aβ (Aβ; **E**, **F**), ubiquitin (**G**, **H**), glial fibrillary acidic protein (GFAP; **I**, **J**) and Gallyas silver stain counterstained with eosin (**K**, **L**). Note that prominent accumulation of Aβ (**F**) and ubiquitin (**H**), along with astrogliosis (**J**) and abundant silver staining (**L**), occurs exclusively in CatD-KO mice, primarily in the same cells that show high levels of CatD expression in WT mice (**C**). Scale bar (**L**) represents 500 μm and is applicable to images in **A**-**L**. Quantification of each staining (except H&E) in different brain regions is provided in Sup Fig. 2. **M**-**S**, Comparative analysis of Gallyas silver staining in a 3-week-old CatD-KO mouse (**M**-**Q**) versus a 77-year-old Alzheimer disease (AD) human brain (**R**, **S**). Note how closely Gallyas silver staining in mouse neurons (**Q**) resembles the staining of NFTs in human neurons (**R**), with both featuring prominent perinuclear staining (white arrowheads). Scale bars are 1 mm (**M**), 500 μm (**N**), 100 μm (**O**), 50 μm (**P**, **S**) and 20 μm (**Q**, **R**)

The profound intracellular amyloid accumulation triggered by deletion of CatD raised the question of whether other pathological hallmarks of AD might be present in CatD-KO mice, prompting us to perform additional immunohistochemical characterization. To simplify interpretation (and because subsequent discoveries required different tissue collection procedures not used for APP+ mice, which had already been harvested), we elected to focus exclusively on the (hAPP-negative) CatD-KO line [21] for this analysis. To that end, we conducted an extensive analysis of hippocampus (Fig. 2 and Sup Fig. 2) and cortex (Sup Fig. 2) in groups of CatD-KO (KO-) and wildtype (WT-) littermate controls probed with a variety of AD-relevant antibodies and histochemical stains. H&E staining revealed that the gross anatomy of 3-week-old KO- brains was largely similar to WT- brains, with the exception of small numbers of pyknotic cells within the hippocampi of a subset of KO- mice (Fig. 2A, B). Staining for CatD protein revealed that the protease is widely expressed in WT- cortex and hippocampus (Fig. 2C), being particularly high within cell bodies throughout the cornu ammonis; as expected, no immunoreactivity was detected in KO- brain (Fig. 2D). As reported previously [9], KO- mice exhibited extensive Aβ immunoreactivity (Fig. 2F); notably, endogenous Aβ deposition overlapped remarkably closely with the regions normally expressing CatD (Fig. 2C), while being essentially undetectable in WT- mice (Fig. 2E and Sup Fig. 2A). Ubiquitin immunoreactivity, a reliable marker of several pathological lesions in AD and other neurodegenerative diseases [43], was prominent in KO- mice (Fig. 2G and Sup Fig. 2B), but not WT- controls (Fig. 2H), once again in a pattern overlapping endogenous CatD expression (Fig. 2C). Staining for astrocytes with glial fibrillary acidic protein (GFAP) revealed extensive astrocytosis in KO- brain (Fig. 2J), in marked contrast to the modest staining present in WT- mice (Fig. 2I and Sup Fig. 2C). KO- and WT- brain sections were also probed with Gallyas silver stain, a widely-used histological marker of NFTs characteristic of AD and other neurodegenerative disorders [44]. KO- mice exhibited an unanticipated and very substantial degree of Gallyas-positive staining (Fig. 2L), yet again overlapping with CatD expression in WT- mice (Fig. 2C), in marked contrast to the relatively low level of staining in WT- controls (Fig. 2K and Sup Fig. 2D). Subsequent analysis at higher resolutions (Fig. 2M-Q) revealed that the Gallyas staining in KO- mice manifested as intense, highly localized, perinuclear staining that resembled—to a remarkable extent—Gallyas-positive NFTs present in AD brain (Fig. 2R, S; cf. Figure 2Q and R).

**Fig. 3:**
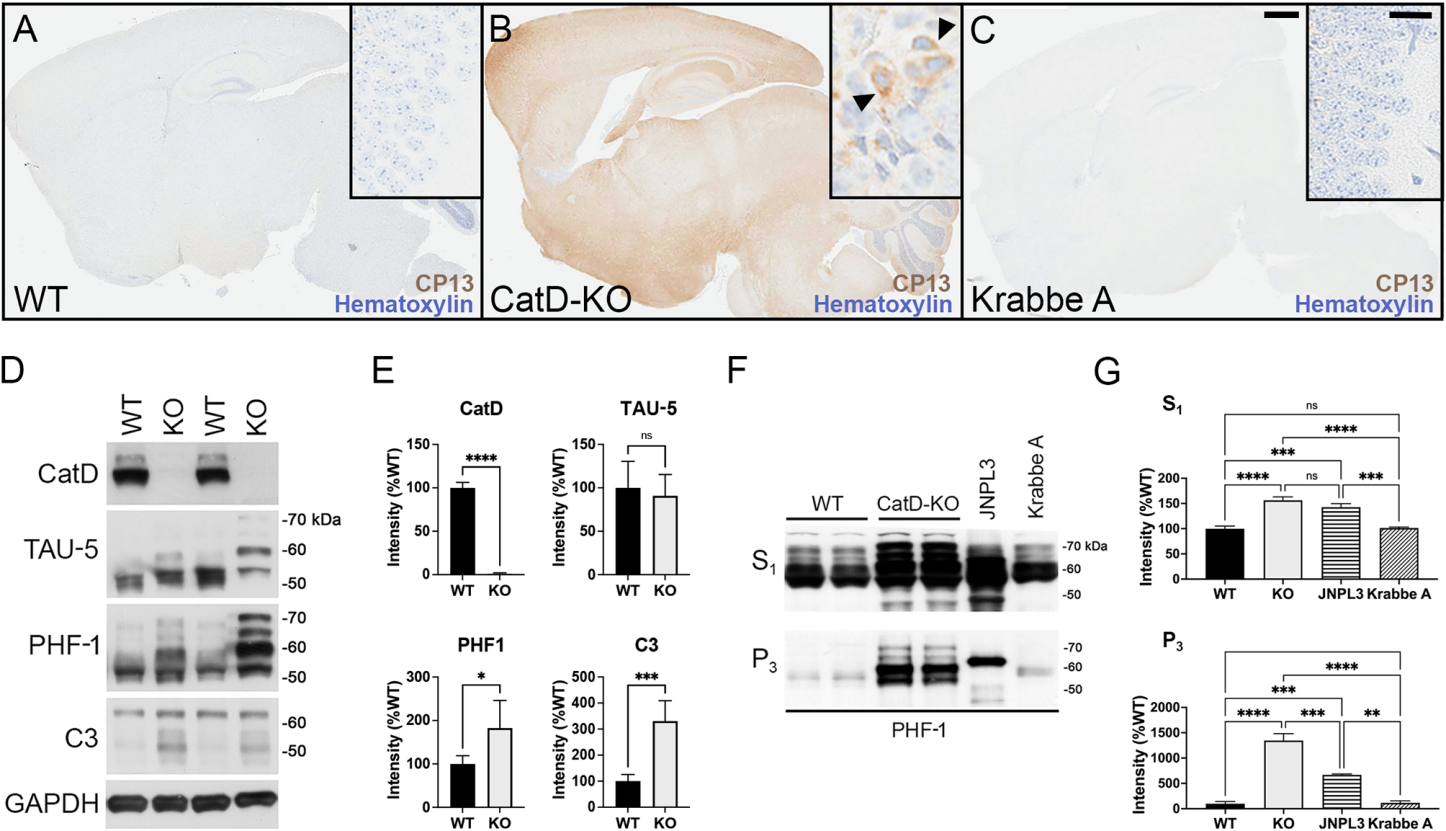
Three-week-old CatD-KO mice exhibit prominent, widespread tauopathy. (**A**-**C**) Sagittal brain sections of wildtype (WT; **A**), CatD-KO (KO; **B**), and Krabbe A (**C**) mice stained for hematoxylin and the phospho-tau-specific antibody CP13. Note the abundant CP13 immunoreactivity evident in CatD-KO mice but lacking in WT or Krabbe A controls. High-magnification images of hippocampal CA3 regions (insets) reveal prominent perinuclear phospho-tau staining in CatD-KO mice (**B**, arrowheads), resembling the localization of Gallyas silver staining seen in Fig. 2. Scale bars (**C**) are 1 mm for all main images and 50 μm for all insets. (**D**, **E**) Western blotting (**D**) and quantitation thereof (**E**) in ∼ 3-week-old WT and KO mice (*n* = 5 per genotype), showing levels of cathepsin D (CatD), total tau (TAU-5), phospho-tau (PHF-1) and caspase-cleaved tau (C3) along with GAPDH as a loading control. Note that total tau levels are not increased quantitatively (**E**, upper right graph), despite the pronounced perturbations in the migration pattern of total tau in CatD-KO mice consistent with hyperphosphorylation. (**F**, **G**) Representative PHF-1 western blots (**F**) and quantitation thereof (**G**) of soluble (S_1_) and sarkosyl insoluble (P_3_) brain extracts from ∼ 3-week-old wildtype (WT) and CatD-KO mice (*n* = 4 per genotype) together with 9-month-old hTau transgenic (JNPL3) and 3-month-old Krabbe A controls (*n* = 2 per genotype). Note that CatD-KO mice harbor levels of sarkosyl-insoluble tau by just 3 weeks of age exceeding those present in 9-month-old JNPL3 mice, which show abundant tauopathy by this age [26]. Note further that Krabbe A mice, which feature marked lysosomal disturbances and were collected at a similar antemortem interval as the CatD-KO mice, show no increases in soluble or sarkosyl-insoluble tau relative to WT mice (**F**, **G**). Data are mean ± SEM; *P < 0.05; **P < 0.01; ***P < 0.001; ****P < 0.0001; ns = not significant

The discovery of strong Gallyas-positive staining in CatD-KO mice reminiscent of mature NTFs in AD brain, though not itself a tau-specific marker, inspired us to investigate other tau-related endpoints. Staining with the phospho-Ser202-specific anti-tau antibody CP13 [31] revealed intense phospho-tau staining present throughout KO- brain (Fig. 3B), but essentially absent from WT- brain (Fig. 3A). Notably, as was true for Gallyas staining, CP13 immunoreactivity in KO- mice was frequently present in a perinuclear localization pattern (Fig. 3B, inset). Crucially, to control for potential influences of generic lysosomal dysfunction or non-specific antemortem agonal conditions in the KO- mice, we also analyzed CP13 staining in twitcher mice [24], a model of Krabbe A disease attributable to galactocerebrosidase (*GALC*) deficiency (referred to here as Krabbe A mice). Like CatD-KO mice, Krabbe A mice feature both profound lysosomal disturbances as well as premature lethality occurring at a reliably predictable age [24]. Krabbe A mouse brains, which were harvested at an identical antemortem interval as CatD-KO mice (∼ 2–3 days) and processed and stained for CP13 in parallel showed virtually no CP13 immunoreactivity, being indistinguishable from CatD-WT mice (Fig. 3C). Additional staining of KO- and WT- brains with CP13 and the phospho-Ser396/404-specific anti-tau antibody, PHF-1 [32], conducted on serial sections, yielded substantially similar results (Sup Fig. 3A, B). To further confirm the latter results, we conducted additional staining with the phospho-Thr231-specific anti-tau antibody, AT180 [33], in this case conducted in parallel with JNPL3 hTau transgenic mice [26] and AD brain as positive controls (Sup Fig. 4A-H). AT180 immunoreactivity appeared substantially similar to both CP13 and PHF-1 staining, being essentially absent in WT brains (Sup Fig. 4A, B) but widespread throughout CatD-KO brain (Sup Fig. 4C, D). As expected, AT180 staining in JNPL3 mice and AD brain was characterized by intense perinuclear staining confined to a subset of isolated cells in hippocampus and cortex (Sup Fig. 4E-H); a similar pattern was observed in CatD-KO brains, with a subset of neuronal cells, including dentate gyrus mossy cells, showing intense perinuclear AT180-immunoreactivity, albeit on a background of relatively ubiquitous phospho-tau staining (Sup. Figure 4C, D). Quantitation of phospho-tau staining by all 3 antibodies is provided in Sup Fig. 5A-C. Finally, though not considered an especially specific marker, it is noteworthy that thioflavin S fluorescence was elevated > 75-fold in KO- brain relative to WT- brain but was not significantly increased in Krabbe A brain (Sup Fig. 6A-E).

To more fully explore the consequences of CatD deletion on tau abundance and phosphorylation status, we performed western blotting on whole-brain extracts from KO- and WT- mice specifically prepared to preserve protein phosphorylation. No significant differences in total tau protein levels were detected between KO- and WT- brains, as ascertained from western blotting with the TAU-5 antibody (Fig. 3D, E). However, marked changes in the migration of tau were evident in extracts from KO- mice even from total-tau staining, manifesting in the form of multiple tau species electrophoresing significantly more slowly in KO- extracts versus WT- controls (Fig. 3D). Western blotting for phospho-tau species with PHF-1 confirmed that the abnormally migrating tau species are indeed hyperphosphorylated and also revealed increases in phospho-tau overall in KO- versus WT- brains (Fig. 3E). Also, confirming previous reports [17], CatD-KO mice exhibited highly significant elevations in a C-terminally truncated, caspase-cleaved form of tau strongly implicated in NFT formation in AD [45] (Fig. 3D, E).

The definitive biochemical hallmark of NFT formation is the accumulation of insoluble hyperphosphorylated tau species, specifically sarkosyl-insoluble forms [3]. Using established protocols [46], we prepared soluble (S_1_) and sarkosyl-insoluble (P_3_) brain extracts from WT- and KO- mice and probed them by western blotting with PHF-1. As a positive control, we also analyzed JNPL3 hTau transgenic mice, which develop abundant tauopathy beginning at 6 months of age [26]. Relative to WT- controls, KO- mice exhibited profound ∼ 1,250% increases in sarkosyl-insoluble, PHF-1-positive phospho-tau by just ∼ 3 weeks of age that—remarkably—greatly exceeded the levels in 9-month-old JPNL3 mice [26] (Fig. 3F, G). PHF-1-positive phospho-tau levels were also significantly increased in the soluble fraction, though to a lesser extent (Fig. 3F, G). Importantly, extracts from Krabbe A mice harvested at a similar antemortem interval and processed in parallel were essentially indistinguishable from WT- mice (Fig. 3F, G). Together, these biochemical findings demonstrate that CatD dysfunction can trigger profound tauopathy in vivo, independently of generic lysosomal impairments.

**Fig. 4:**
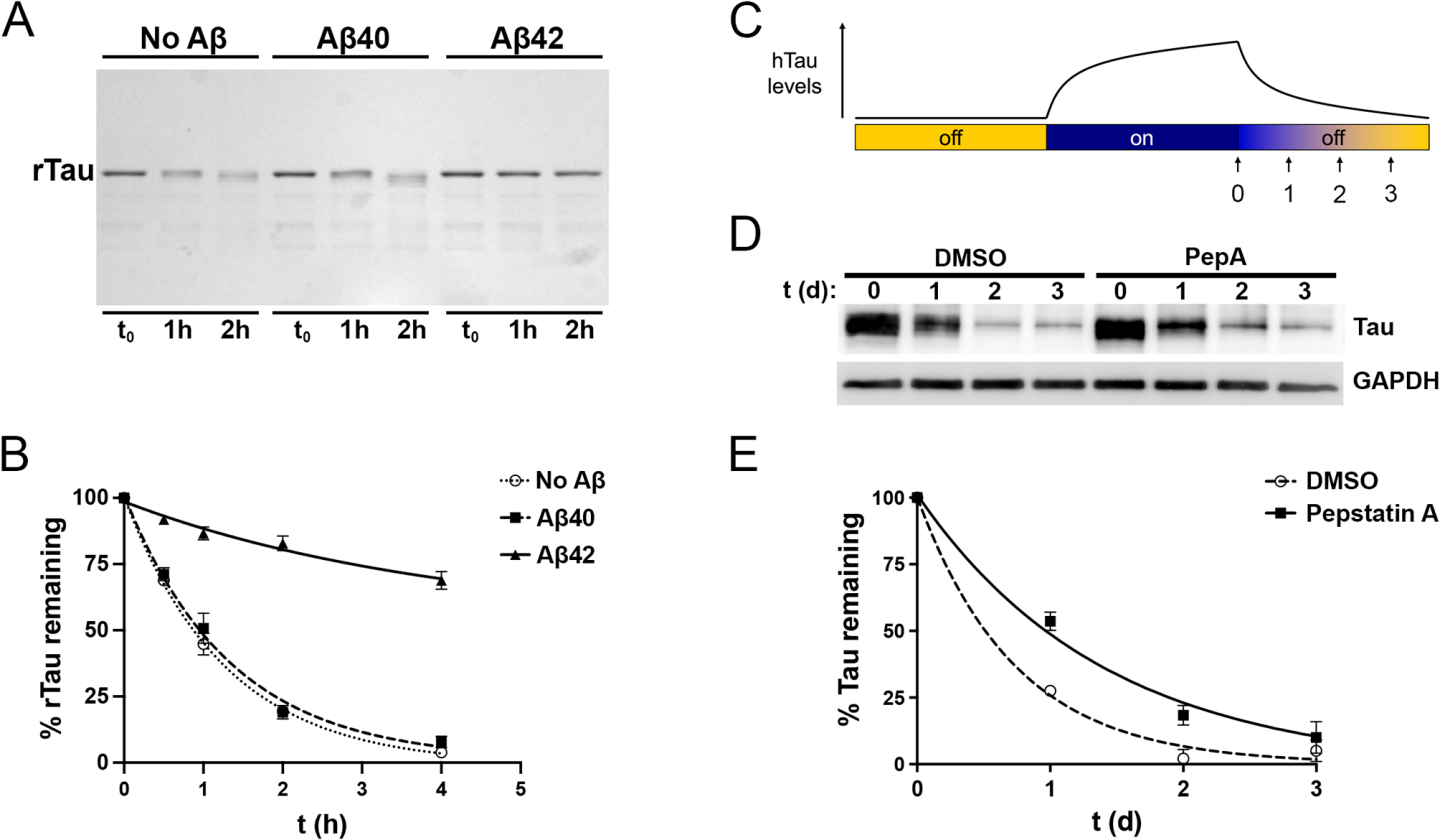
Inhibition of CatD slows the catabolism of tau in vitro and in cultured cells. (**A**) Representative Coomassie blue-stained polyacrylamide gel loaded with recombinant human tau (rTau) incubated for the indicated times with recombinant human CatD (5 nM) in the absence or presence of equal concentrations (1 µM) of Aβ40 or Aβ42. (**B**) Quantification of rTau levels as a function of time in 4 independent experiments. Note how rTau catabolism is unaffected by Aβ40 but markedly slowed by Aβ42, a potent competitive inhibitor of CatD. Data are mean ± SEM; *n* = 4. (**C**) Overview of the experimental approach used to quantify hTau catabolism in “Tet-Off” cultured neuroblastoma cells (see main text). (**D**) Representative western blot showing hTau levels (stained with antibody P44) at different time points after cessation of hTau expression in the absence or presence of the CatD inhibitor, pepstatin A (PepA; 1 µM). (**E**) Quantitation of hTau levels as a function of time from 6 independent experiments. Note the marked increase in the half-life of hTau in the presence of PepA (0.98 days; 95% CI 0.80 to 1.25) relative to DMSO-treated controls (0.51 days; 95% CI 0.429 to 0.627; *P* = 0.0012). Data are mean ± SEM, *n* = 6

To extend and refine these in vivo findings, we investigated tau catabolism in two experimental paradigms wherein CatD proteolytic activity, rather than CatD protein levels, was selectively manipulated. We first established a simple in vitro paradigm, wherein the catabolism of recombinant human tau (rTau) directly by purified human CatD could be monitored via Coomassie blue staining of rTau run on polyacrylamide gels (Fig. 4A). Based on our prior work establishing that Aβ42, but not Aβ40, inhibits CatD with subnanomolar potency [9], we monitored CatD-mediated rTau catabolism in the absence or presence of identical concentrations (1 µM) of freshly prepared, SEC-purified, monomeric human Aβ40 or Aβ42 [38, 47]. As reported previously for several other CatD substrates [9], Aβ42 strongly inhibited rTau degradation by CatD, whereas an identical concentration of Aβ40 exerted essentially no effect (Fig. 4A, B and Sup Table S1).

To assess potential effects of CatD proteolytic activity on human tau (hTau) catabolism in living cells, we studied the M1C cell model, a human neuroblastoma (BE(2)-M17D) cell line that expresses the human 4R0N tau isoform in a tetracycline (Tet)-dependent (Tet-Off) manner [36]. Catabolism of hTau was monitored as follows (see Fig. 4C). After growing M1C cells for 4 days in the absence of Tet to permit maximal hTau expression, Tet (2 µg/mL) was added to suppress hTau expression, then cells were harvested for protein extraction 0, 1, 2 and 3 days later (Fig. 4C). hTau levels in cell lysates were then monitored by western blotting with the anti-hTau antibody, P44 (see Fig. 4D). To test whether CatD proteolytic activity might impact hTau catabolism, we monitored hTau levels as a function of time in the absence or presence of pepstatin A (PepA; 1 µM), a highly potent (IC_50_ < 0.1 nM) inhibitor of CatD [48]. Based on 6 independent experiments, the half-life of hTau in the presence of PepA (0.98 days; 95% CI 0.80 to 1.25) was found to be approximately double that in DMSO-treated controls (0.51 days; 95% CI 0.429 to 0.627) in this system, a statistically significant increase (*P* = 0.0012; Fig. 4E).

## Discussion

We report here that genetic deletion of the lysosomal protease CatD triggers the development of the two principal proteinopathies specifically pathognomonic for AD: Aβ accumulation and tauopathy. The Aβ accumulation in CatD-KO mouse brain is notable in several respects, each meriting discussion. First, analysis of hAPP transgenic mice confirmed that Aβ does in fact accumulate as a result of CatD deletion and is not an artifact of anti-Aβ antibodies interacting nonspecifically with lipofuscin, as suggested by some early studies [40–42]. Indeed, to the contrary, our findings suggest that these studies might need to be reinterpreted as demonstrating that Aβ does in fact accrue within compartments that also accumulate lipofuscin in AD. Second, Aβ accumulates exclusively intracellularly in both KO + and KO- mouse brains, in the absence of extracellular deposition [9]. This result implies that a substantial portion of Aβ is normally trafficked to lysosomes, which is sensible given that the β- and 𝛾-secretases responsible for producing Aβ are both aspartyl proteases most active within acidic compartments of the endolysosomal pathway [49]. Similarly, the lack of effect of CatD deletion on extracellular deposits comports with the fact that CatD is an aspartyl protease that is essentially inactive toward Aβ degradation under the neutral conditions present extracellularly [9, 14]. Consistent with this, intracranial infusion of recombinant pro-CatD, which is subsequently converted to active CatD, was recently shown to exert no effect on extracellular amyloid deposition in an AD mouse model [20]. Finally, corroborating previous reports [9, 18], we find no evidence that steady-state Aβ levels or amyloid plaque formation are affected by haploinsufficiency of CatD, neither in the absence nor in the presence of hAPP overexpression. Thus, by contrast to most other Aβ-degrading proteases [50, 51], CatD is not a rate-limiting regulator of Aβ in vivo, consistent with the idea that the lysosome represents a high-capacity sink for the clearance of Aβ, as it appears to be for many other substrates [52]. This result in turn implies that intralysosomal Aβ accumulation can occur if and only if CatD activity and/or levels drop below some threshold level, apparently well below 50% of wildtype levels.

The most significant outcome of the present study is the discovery that CatD-KO mice develop widespread, robust tauopathy by just ∼ 3 weeks of age, as evident from several independent measures, including Gallyas silver staining resembling NFTs in AD brain, phospho-tau immunoreactivity using multiple well-characterized antibodies, and western blotting for sarkosyl-insoluble phospho-tau. Because tau is predominantly a cytosolic protein, it is unsurprising that we detected no significant effects on overall tau levels in CatD-KO mice. How, though, might the lysosomal protease CatD interact with tau in a manner that could increase phospho-tau? Although the precise mechanisms remain to be established, accruing evidence has demonstrated that a subset of tau protein can be trafficked to the lysosome via several distinct pathways [23]. Among several identified trafficking mechanisms, endogenous tau can be secreted via unconventional protein secretion pathways from primary neurons [53–58] and, once secreted, re-enter neurons and other cell types via fluid-phase endocytosis and micropinocytosis [56], whereupon it can be trafficked to the lysosome via conventional mechanisms. In addition, tau can also enter the lysosome directly from the cytosol, either via macroautophagy [59] or via a selective pathway known as chaperone-mediated autophagy (CMA), wherein substrate proteins directly cross from the cytosol into the lysosome [60]. CMA is mediated by a specific targeting motif (KFERQ-like), present within tau, that binds to the cytosolic chaperone, HSC70, which then brings the substrate to the lysosomal surface for internalization [61]. These trafficking pathways identify lysosomes as a critical locus where tau and CatD (and Aβ) can interact, and our findings are consistent with the idea that phospho-tau species might be preferentially trafficked to lysosomes, where they are normally degraded by CatD, but we emphasize that the specific mechanism(s) by which CatD deficiency triggers increases in phospho-tau remain to be elucidated.

To our knowledge, only two other studies have investigated the consequences of CatD deletion on tau-related endpoints in vivo, and it is instructive to compare these findings to our own. In a fly model overexpressing mutant hTau in eye, deletion of CatD markedly exacerbated mutant hTau-induced pathology and premature lethality [17]. Consistent with our own results, this study found that total tau levels were not increased by CatD deletion in flies or mice [17]. This study also found that a C-terminally truncated, caspase-cleaved form of tau found in AD patients was significantly increased in both models, as we confirm here [17]. Further corroborating our own results, a second, recent study found that neuron-specific deletion of *CTSD* triggers robust phospho-tau immunoreactivity, albeit in only a subset of neurons [62].

Although reaffirming our own findings, neither of the aforementioned studies detected the striking degree of sarkosyl-insoluble phospho-tau we observed in CatD-KO mice. The magnitude of the tau pathology resulting from deletion of CatD is notably not only because it exceeds the tauopathy present in a robust mouse model of tauopathy, but also because it occurs by just ∼ 3 weeks of age. Moreover, the finding that tau pathology was entirely absent from a mouse model of a different lysosomal disease, Krabbe A, one that also suffers premature lethality, lends strong support to the idea that these changes are specific to CatD deficiency and not attributable to non-specific effects of lysosomal dysfunction or antemortem agonal conditions. Similarly, no previous studies detected Gallyas-positive perinuclear inclusions found in CatD mice, which are strikingly reminiscent of argyrophilic staining of mature NFTs characterizing AD. To our knowledge, CatD-KO mice represent the only animal model to develop such marked tauopathy in the absence of overexpression of hTau harboring disease-linked mutations [19].

The simultaneous accrual of both Aβ and hyperphosphorylated tau resulting from CatD deletion is particularly noteworthy when considered together with our earlier discovery that CatD proteolytic activity is inhibited specifically and extremely potently by Aβ42 [9, 23], the Aβ species most strongly linked to AD pathogenesis [2]. Not only was Aβ42 established to be a subnanomolar competitive inhibitor of CatD, but this length-specific inhibitory effect was also shown to extend to shorter Aβ species ending at position 42 [9], including the ⍺-secretase-derived P3 fragment of APP that is generated ∼ 10-fold more abundantly than Aβ42 [22]. We show here that inhibition of CatD proteolytic activity with Aβ42 or PepA slows the catabolism of hTau in vitro and in cultured cells, respectively. Taken together, these findings support the hypothesis that Aβ42 accumulation may contribute to tau pathology (and potentially other pathological sequelae) in part via proteolytic inhibition of CatD [23]. This hypothesis predicts that tauopathy should emerge in a cell-autonomous fashion, with cells harboring severe tauopathy capable of arising even in close proximity to unaffected cells—and this is indeed how tauopathy manifests in human AD (see Sup Fig. S4G, H) [63].

The principal limitations of the present study stem from the premature lethality triggered by germline genetic deletion of *CTSD*, which precludes analysis of age-related pathology. Mouse models permitting conditional deletion of *CTSD* have been developed [62, 64], and as mentioned, one model featuring neuron-specific deletion also showed some evidence of tau pathology [62]; unfortunately, deletion of CatD exclusively in neuroectoderm [64] or neurons [62] also results in premature lethality. There is a great need, therefore, to develop improved animal models that permit the manipulation of CatD inducibly and/or more flexibly. To that end, our group recently developed a novel experimental approach, dubbed “TRE-Lox,” that permits *CTSD* to be alternatively irreversibly deleted, through conventional CreLox technology, or downregulated as much as 98% in a completely reversible, doxycycline-regulatable manner [65]. We are currently in the process of making mouse lines implementing this system, which we anticipate will help further elucidate the role(s) of CatD in the pathogenesis of AD and other neurodegenerative diseases.

## Conclusions

Our results strongly implicate CatD in the proteostasis of both Aβ and tau in vivo, suggesting that deficiencies in CatD levels and/or activity might play a causal role in the pathogenesis of AD and potentially other tauopathies. This is significant in light of accruing genetic evidence linking a common loss-of-function mutation in *CTSD* to AD risk [5–8] and other evidence linking impairments in CatD to multiple neurodegenerative disorders [10–12]. To our knowledge, the CatD-KO line is the only mouse model that develops both detectable accumulation of endogenous Aβ and robust tauopathy in the absence of overexpression of hAPP or hTau harboring disease-causing mutations. That these proteinopathies accrue by just ∼ 3 weeks of age suggests that CatD dysfunction may play a key role in their etiology. Furthermore, because CatD is potently inhibited by Aβ42 [9], our findings suggest a compelling interrelationship among intraneuronal Aβ accumulation, CatD deficiency and tauopathy manifesting at the level of the lysosome. Given that lysosomal dysfunction is a common feature in multiple age-related neurodegenerative diseases [66], additional research into this interrelationship is clearly warranted.

### Electronic supplementary material

Below is the link to the electronic supplementary material.

Supplementary Material 1

Supplementary Material 2

## Data Availability

All data generated or analyzed during this study are included in this published article and its supplementary information files.

## Abbreviations

Aβ: Amyloid-β protein
AD: Alzheimer disease
CatD: Cathepsin D
*CTSD*: Cathepsin D gene
CMA: Chaperone-mediated autophagy
DAB: 3,3’-diaminobenzidine
*GALC*: Galactocerebrosidase gene
GFAP: Glial fibrillary acidic protein
H&E: Hematoxylin and eosin
HET: Heterozygous null
hAPP: Human amyloid precursor protein
hTau: Human tau
rTau: Recombinant human tau
KO: Knockout
NFTs: Neurofibrillary tangles
PepA: Pepstatin A
Tet: Tetracycline
WT: Wildtype
