# Supplementary material for: Prominent tauopathy and intracellular β-amyloid accumulation triggered by genetic deletion of cathepsin D: implications for Alzheimer disease pathogenesis
Source: Alzheimers Res Ther. 2024 Apr 4;16:70. doi: 10.1186/s13195-024-01443-6 (PMC10996108; doi:10.1186/s13195-024-01443-6)
Supplement: Supplementary file 1 — Supplementary Material 1 [file 13195_2024_1443_MOESM1_ESM.docx]

**Supplementary Information for**

**Prominent tauopathy and intracellular β-amyloid accumulation triggered by genetic deletion of cathepsin D: Implications for Alzheimer disease pathogenesis**

Heather M. Terron^1^, Sagar J. Parikh^1^, Samer O. Abdul-Hay^2^, Tomoko Sahara^2^, Dongcheul Kang^2^,

Dennis W. Dickson^2^, Paul Saftig^3^, Frank M. LaFerla^1,4^, Shelley Lane^1^, and Malcolm A. Leissring^1,2,*^

^1^ Institute for Memory Impairments and Neurological Disorders, University of California, Irvine (UCI MIND), Irvine, CA 92697, USA

^2^ Department of Neuroscience, Mayo Clinic Florida, Jacksonville, FL 32224, USA

^3^ Institut für Biochemie, Christian-Albrechts-Universität zu Kiel, D-24098 Kiel, Germany

^4^ Department of Neurobiology and Behavior, University of California, Irvine, Irvine, CA 92697, USA

**Contents**

**Page Figure Title .**

**2 Sup Fig. 1 Aβ plaque burden in older APP+ mice and Aβ levels in older APP- mice.**

**3 Sup Fig. 2 Quantitation of immunohistochemical staining for AD-relevant markers in WT- and KO- mice.**

**4-5 Sup Fig. 3 Immunostaining for CP13 and PHF-1 in WT- and KO- mice.**

**6-7 Sup Fig. 4 Immunostaining for AT180 in WT- and KO- mice using JNPL3 hTau transgenic mice and AD brain as positive controls.**

**8 Sup Fig. 5 Quantitation of immunostaining in WT- and KO- mice by anti-phospho-tau antibodies.**

**9 Sup Fig. 6 Thioflavin S fluorescence in WT- , KO- and Krabbe A mice.**

**10 Sup Table 1 Data for individual runs of in vitro rTau catabolism experiments in Fig. 4B.**

**Sup Figure 1**

**
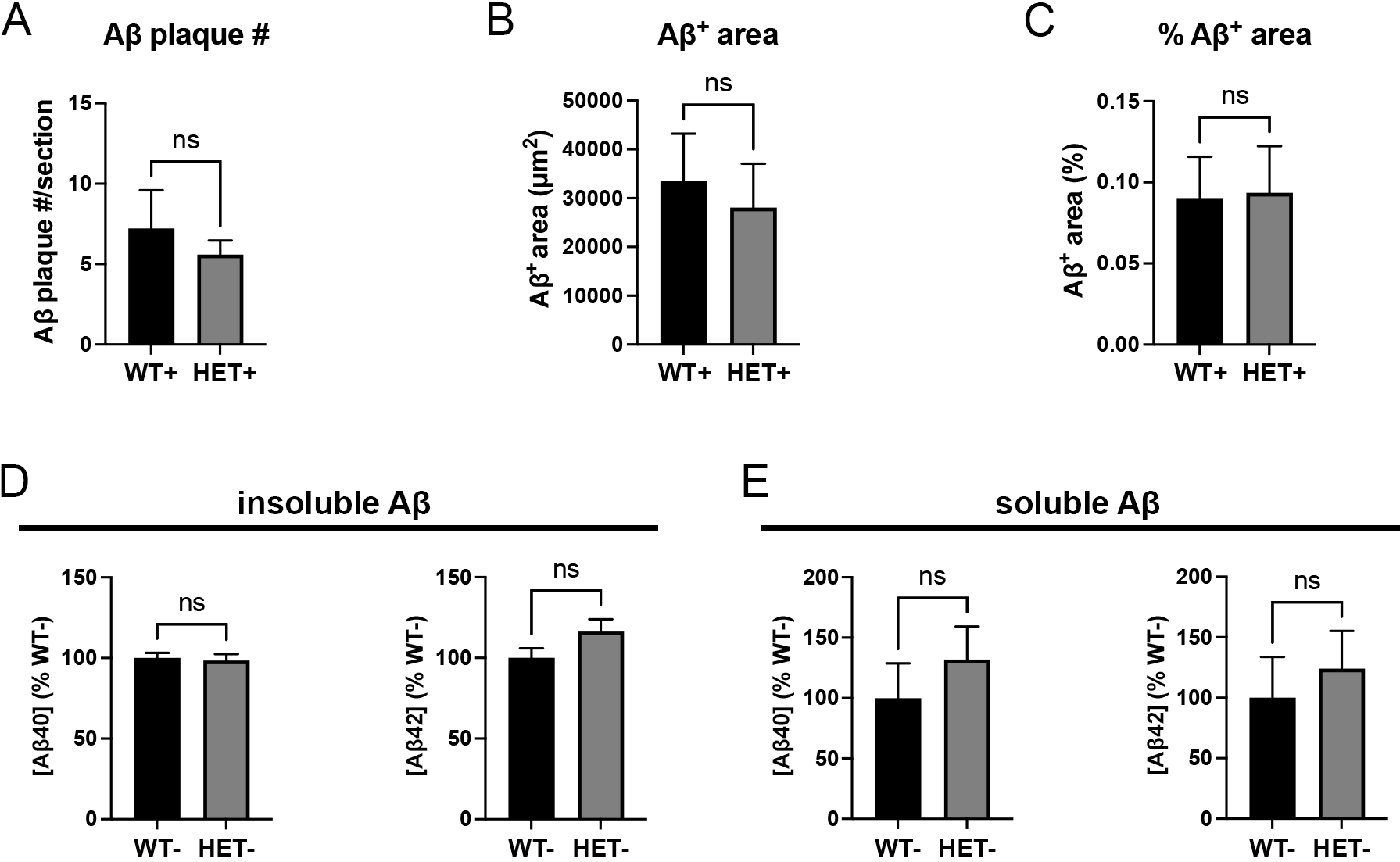
**

**Aβ plaque burden in older APP+ mice and Aβ levels in older APP- mice.** (**A-C**) Amyloid plaque number per section (**A**) and Aβ-positive area expressed in absolute terms (**B**) and as a percentage of total tissue area (**C**). Data are mean ± SEM, n=14-17 brains per genotype. (**D**,**E**) Steady-state levels of insoluble (**D**) and soluble (**E**) endogenous murine cerebral Aβ40 (left) and Aβ42 (right) in 10-month-old WT- and HET- mice. Data are normalized to WT- mice and are expressed as mean ± SEM, n=6 brains per genotype. ns=not significant.

**Sup Figure 2**


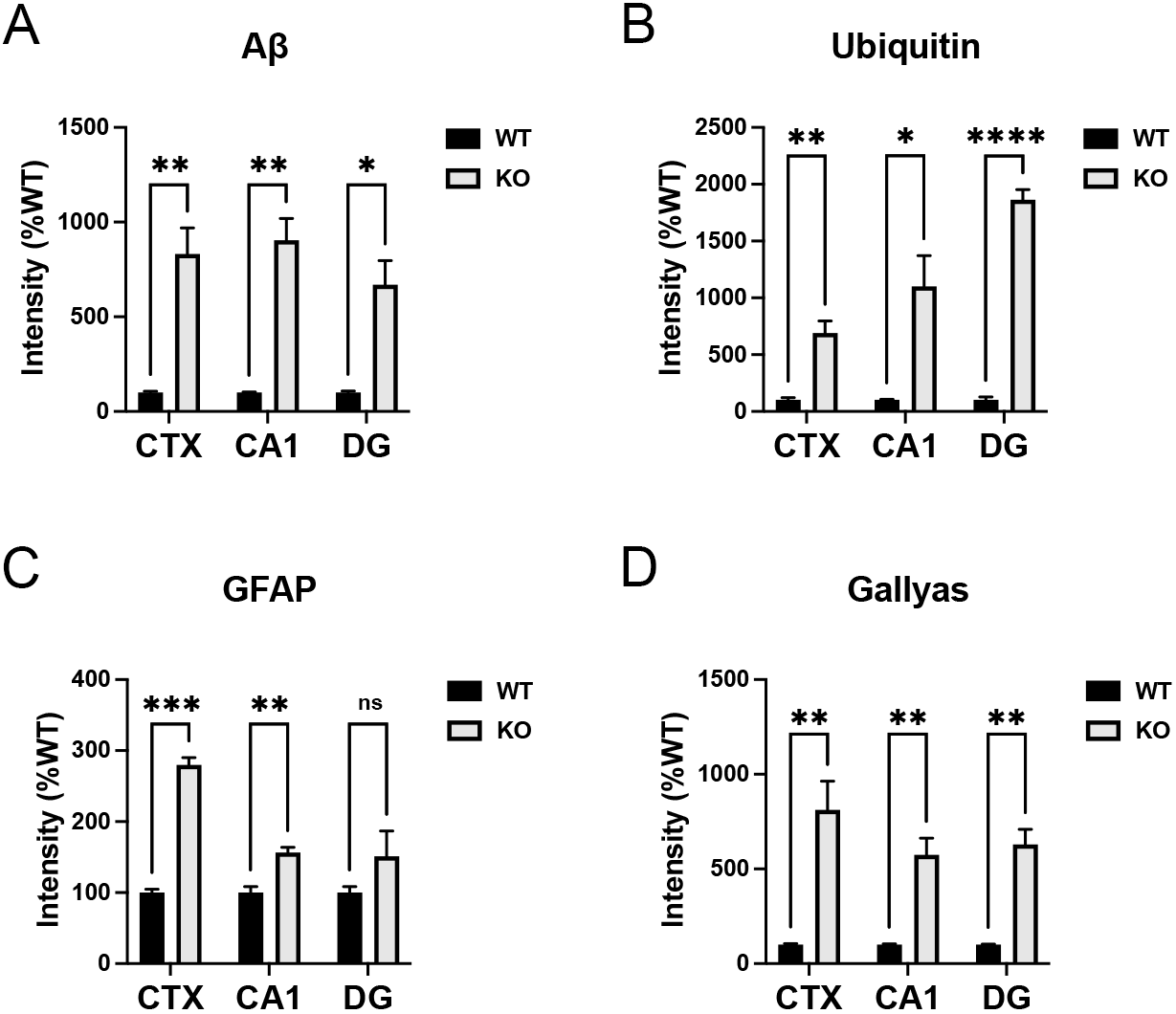


**Quantitation of immunohistochemical staining for AD-relevant markers in WT- and KO- mice.** (**A**-**D**) Relative intensity of immunostaining for total Aβ (**A**), ubiquitin (**B**), and GFAP (**C**), and relative intensity of Gallyas silver staining (**D**) in cortex (CTX) and hippocampal CA1 (CA1) and dentate gyrus (DG) regions of WT- and KO- mouse brain sections determined using QuPath [62]. Data are mean ± SEM, n=3-6 sections per genotype and are normalized to WT- levels for the given brain region. **P*<0.05, ***P*<0.01, ****P*<0.001, *****P*<0.0001, ns=not significant.

**Sup Figure 3**

(figure only; figure legend on next page)

­
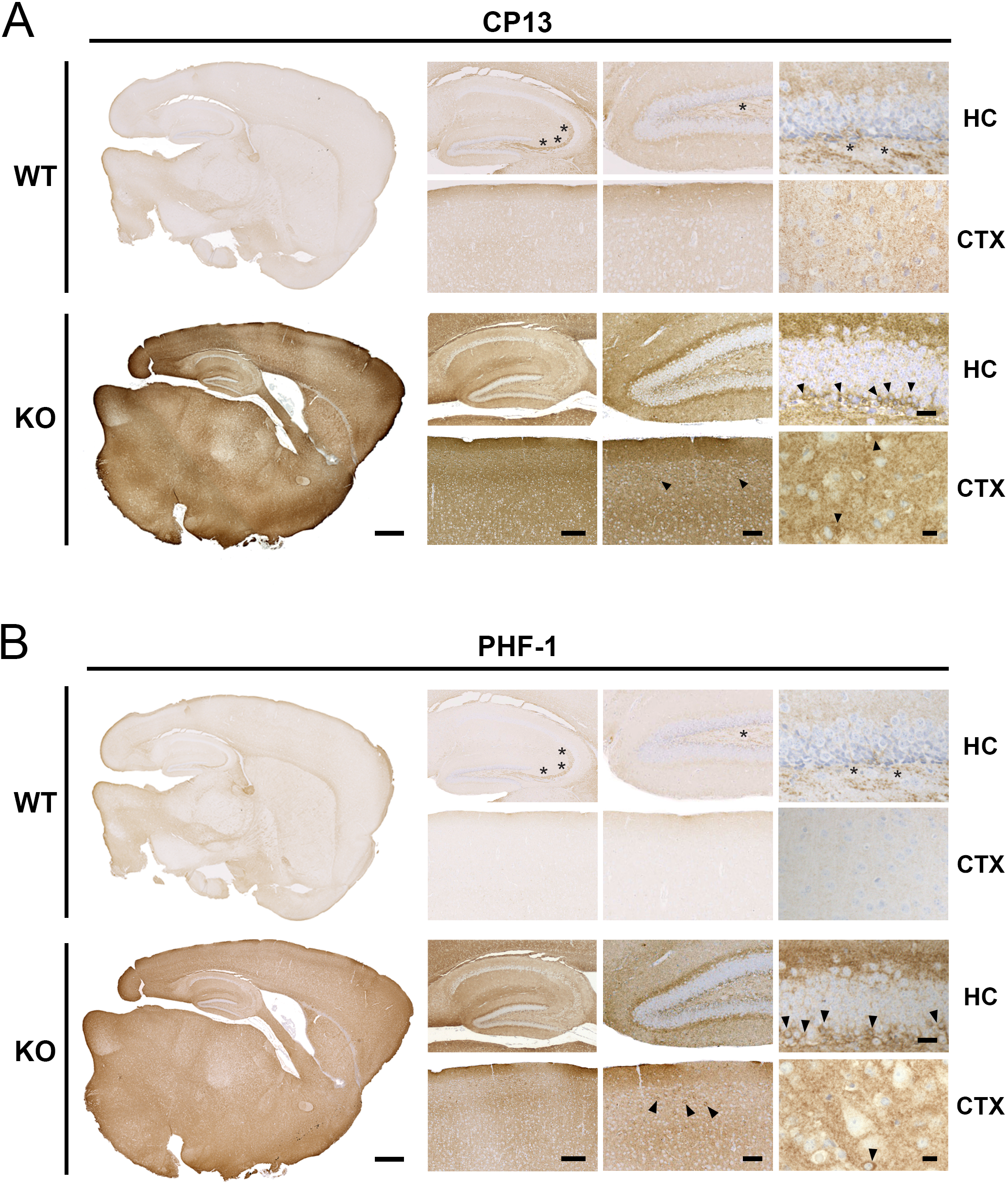


**Sup Figure 3**

(figure legend only; figure on previous page)

**Immunostaining for CP13 and PHF-1 in WT- and KO- mice.** (**A**,**B**) Representative images of WT- and KO- brains stained with the phospho-tau antibody, CP13 (**A**) and PHF-1 (**B**), with higher-magnification images highlighting portions of hippocampus (HC) and cortex (CTX). Note that KO- brains stained for both CP13 (**A**) and PHF-1 (**B**) feature intense perinuclear phospho-tau staining evident at high resolution (arrowheads). Note also that some immunoreactivity present in WT- mice is absent from KO- mice (asterisks). Scale bars are: 1 mm for whole brain sections; 500 µm and 200 µm for the left and middle rectangular panels, respectively; and 100 µm and 20 µm for hippocampus and cortex, respectively, in the right rectangular panels.

**Sup Figure 4**

(figure only; figure legend on next page)

**
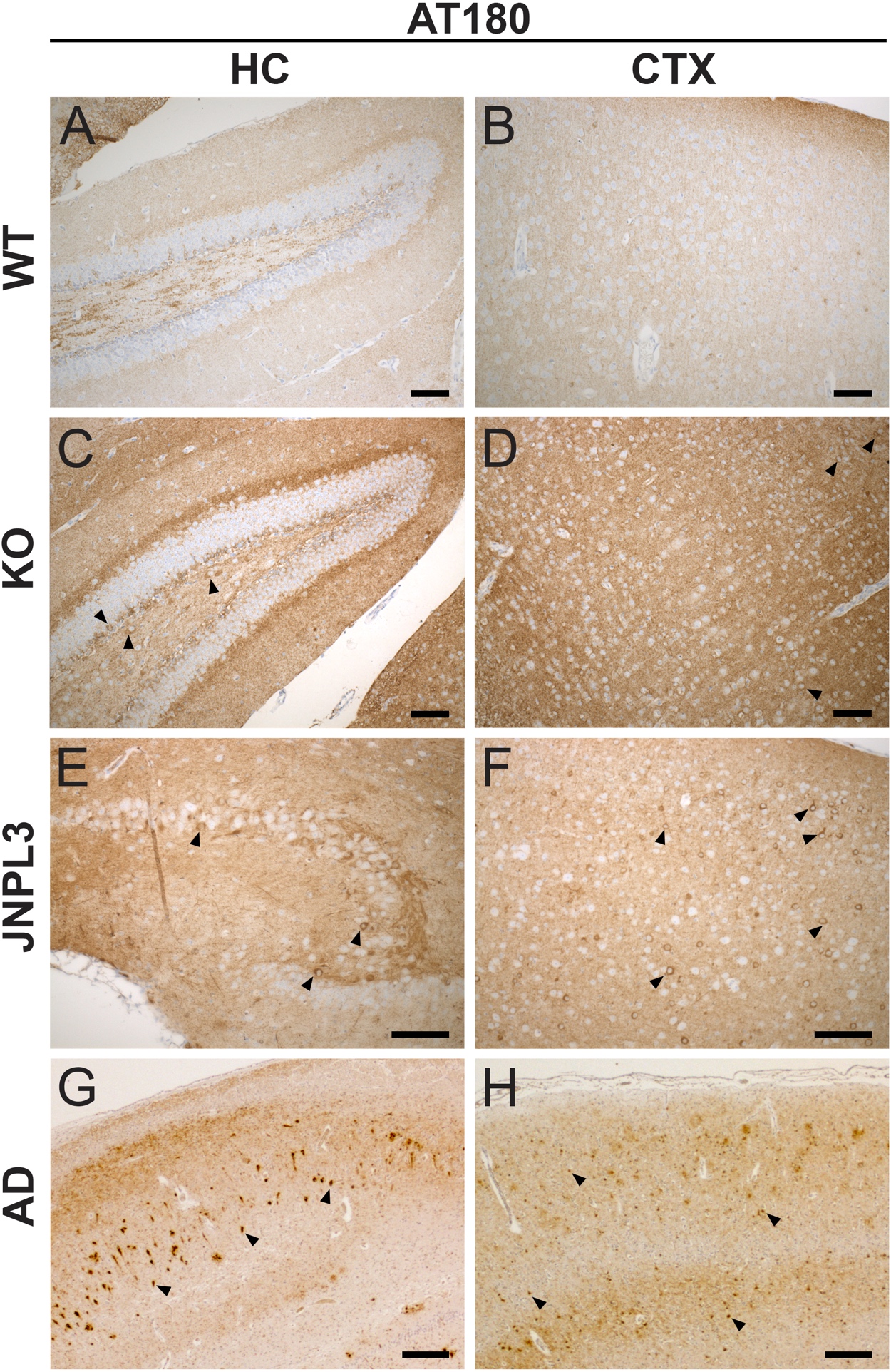
**

**Sup Figure 4**

(figure legend only; figure on previous page)

**Immunostaining for AT180 in WT- and KO- mice using JNPL3 hTau transgenic mice and AD brain as positive controls.** (**A**-**H**) Representative images of hippocampus (HC) (**A**,**C**,**E**,**G**) or cortex (CTX) (**B**,**D**,**F**,**H**) in brains of 3-week-old WT- (**A**,**B**) and KO- (**C**,**D**) mice, 9-month-old JNPL3 hTau transgenic mice (**E**,**F**) and a 77-year-old human patient with Alzheimer's disease (AD) (**G**,**H**) stained with the phospho-Thr231-specific anti-tau antibody, AT180. Note the characteristic presence of isolated neurons staining intensely for phospho-tau (arrowheads) in both the JNPL3 (**E**,**F**) and AD (**G**,**H**) positive controls, a feature that is also evident in a subset of neurons within KO- brains, albeit on a background of widespread, relatively uniform immunostaining (c.f. Sup Fig. 3). Scale bars are: 200 µm for panels **A**-**F** and 500 µm for panels **G** and **H**.

**Sup Figure 5**

**
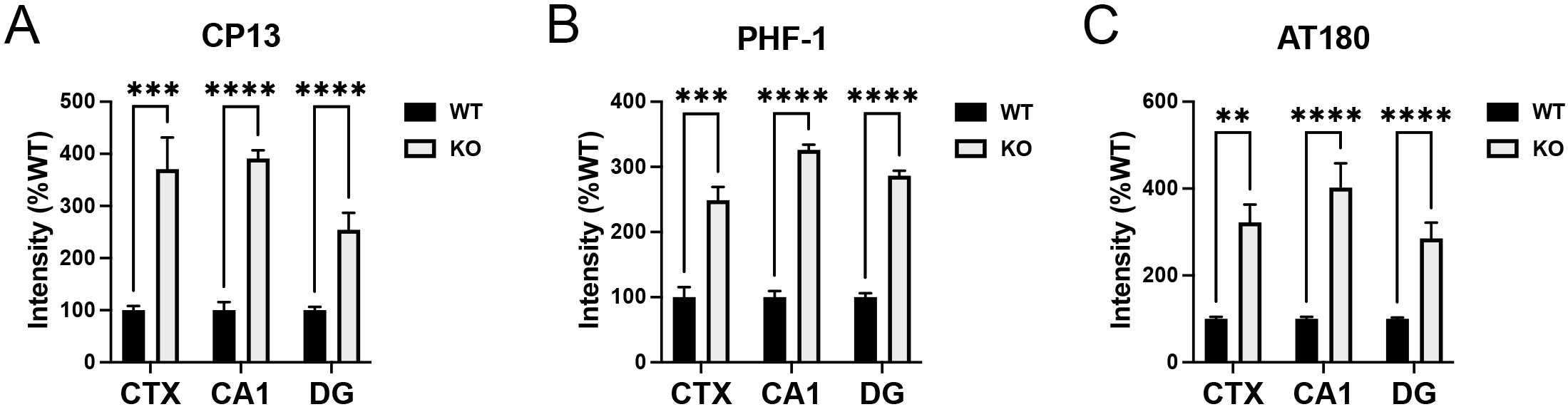
**

**Quantitation of immunostaining in WT- and KO- mice by anti-phospho-tau antibodies.** (**A**-**D**) Relative intensity of immunostaining for total CP13 (**A**), PHF-1 (**B**), and AT180 (**C**) in cortex (CTX) and hippocampal CA1 (CA1) and dentate gyrus (DG) regions of WT- and KO- mouse brain sections determined using QuPath [Ref. 34 in main manuscript]. Data are mean ± SEM, n=3-6 sections per genotype and are normalized to WT- levels for the given brain region. ***P*<0.01, ****P*<0.001, *****P*<0.0001.

**Sup Figure 6**


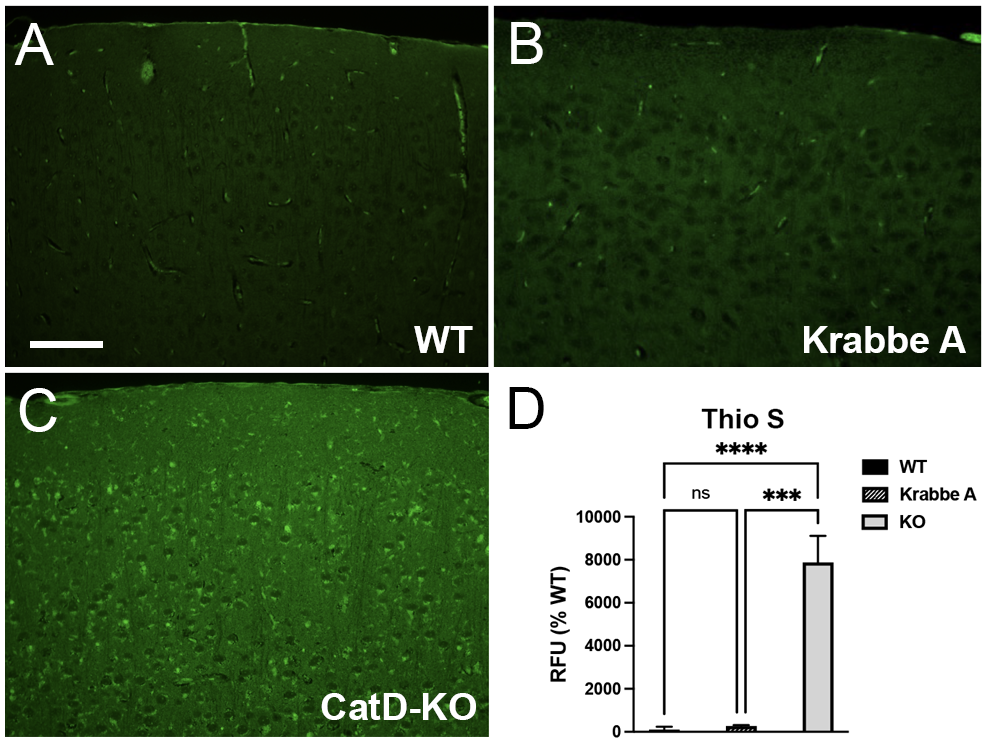


**Thioflavin S fluorescence in WT- , KO- and Krabbe A mice.** (**A**-**C**) Representative images of thioflavin S (Thio S) staining in cortex of 3-week-old WT- (**A**), Krabbe A (**B**) and KO- (**C**) mice. (**D**) Quantitation of relative Thio S fluorescence in WT- and KO- (n=6-7) and 12-week-old Krabbe A (n=3) brains determined from QuPath analysis of fluorescent images (λ_ex_=485 nm, λ_em_=515 nm). Scale bar is 200 µm. Data are mean ± SEM normalized to WT- controls. **P*<0.05; ****P*<0.001; *****P*<0.0001; ns=not significant.

**Sup Table 1**

**Data for individual runs of in vitro rTau catabolism experiments in Fig. 4B.** Data are expressed as percentages of value for sample at t=0 within each condition and experiment.

|  | **t (h)** | **Run 1** | **Run 2** | **Run 3** | **Run 4** |  | **mean** | **SEM** |
| --- | --- | --- | --- | --- | --- | --- | --- | --- |
| **No Aβ** | 0 | 100 | 100 | 100 | 100 |  | 100 | 0 |
|  | 0.5 | 68.9 | 65.5 | 71.7 | 69.6 |  | 68.9 | 1.3 |
|  | 1 | 36.9 | 38.7 | 50.3 | 53.1 |  | 44.8 | 4.1 |
|  | 2 | 17 | 17.7 | 20.1 | 22.7 |  | 19.4 | 1.3 |
|  | 4 | 1.6 | 2.9 | 5.1 | 5.9 |  | 3.9 | 1.0 |
| **Aβ40** | 0 | 100 | 100 | 100 | 100 |  | 100 | 0 |
|  | 0.5 | 73.8 | 67.9 | 76.7 | 66.5 |  | 71.2 | 2.4 |
|  | 1 | 60.7 | 40.8 | 60.8 | 40.9 |  | 50.8 | 5.7 |
|  | 2 | 21.9 | 14.1 | 24.8 | 15.5 |  | 19.1 | 2.6 |
|  | 4 | 13.0 | 4.1 | 10.1 | 4.0 |  | 7.8 | 2.2 |
| **Aβ42** | 0 | 100 | 100 | 100 | 100 |  | 100.0 | 0.0 |
|  | 0.5 | 90.2 | 93.3 | 94 | 90 |  | 91.9 | 1.0 |
|  | 1 | 82.4 | 91.2 | 90.3 | 82.8 |  | 86.7 | 2.4 |
|  | 2 | 77.8 | 88.5 | 86.7 | 78.5 |  | 82.9 | 2.8 |
|  | 4 | 72.7 | 82.7 | 86.6 | 73.7 |  | 78.9 | 3.4 |
