# Supplementary material for: Prominent tauopathy and intracellular β-amyloid accumulation triggered by genetic deletion of cathepsin D: implications for Alzheimer disease pathogenesis
Source: Alzheimers Res Ther. 2024 Apr 4;16:70. doi: 10.1186/s13195-024-01443-6 (PMC10996108; doi:10.1186/s13195-024-01443-6)

Supplementary File

full uncropped images of gels and blots
largest available†
for

Prominent tauopathy and intracellular β-amyloid accumulation triggered by genetic deletion of cathepsin D: Implications for Alzheimer disease pathogenesis

Heather M. Terron[1], Sagar J. Parikh[1], Samer O. Abdul-Hay[2], Tomoko Sahara[2], Dongcheul Kang[2], Dennis W. Dickson[2], Paul Saftig[3], Frank M. LaFerla[1,4], Shelley Lane[1], and Malcolm A. Leissring[1,2,*]

[1] Institute for Memory Impairments and Neurological Disorders, University of California, Irvine (UCI MIND), Irvine, CA 92697, USA

[2] Department of Neuroscience, Mayo Clinic Florida, Jacksonville, FL 32224, USA

[3] Institut für Biochemie, Christian-Albrechts-Universität zu Kiel, D-24098 Kiel, Germany

[4] Department of Neurobiology and Behavior, University of California, Irvine, Irvine, CA 92697, USA

Contents

Page	Title
2	**Figure 3, Panel D, western blot 1 of 5, anti-CatD**
3	**Figure 3, Panel D, western blot 2 of 5, TAU-5**
4	**Figure 3, Panel D, western blot 3 of 5, PHF-1**
5	**Figure 3, Panel D, western blot 4 of 5, C3**
6	**Figure 3, Panel D, western blot 5 of 5, GAPDH**
7	**Figure 3, Panel F, western blot 1 of 2, S_1 fraction, PHF-1**
8	**Figure 3, Panel F, western blot 2 of 2, P_3 fraction, PHF-1**
9	**Figure 4, Panel A, Coommassie-stained polyacrylamide gel**
10	**Figure 4, Panel D, western blot 1 of 2, P44**
11	**Figure 4, Panel D, western blot 2 of 2, GAPDH**

†Please note: The hard drive containing the original images was unfortunately corrupted, and attempts to retreive the data via data recovery services were not successful. The images shown here are the largest available. The authors attest that there are no significant data points outside of these largest-available croppings that would change the interpretation of the results.

Figure 3, Panel D, western blot 1 of 5, anti-CatD

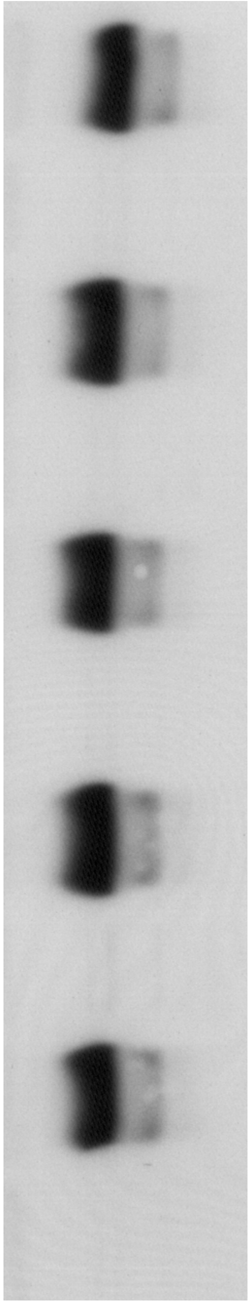

Figure 3, Panel D, western blot 2 of 5, TAU-5

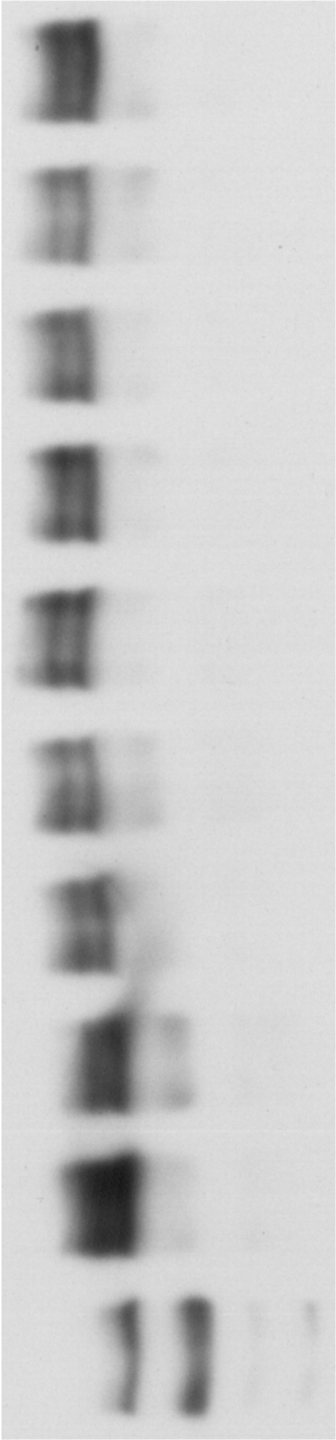

Figure 3, Panel D, western blot 3 of 5, PHF-1

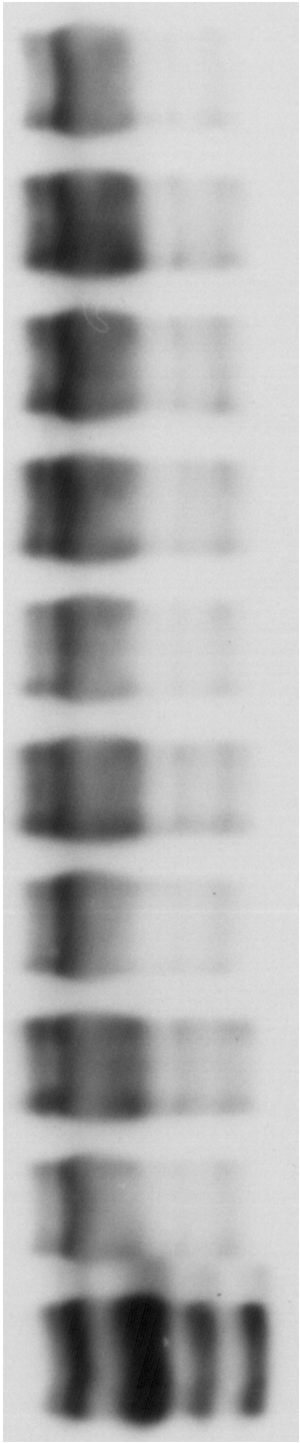

Figure 3, Panel D, western blot 4 of 5, C3

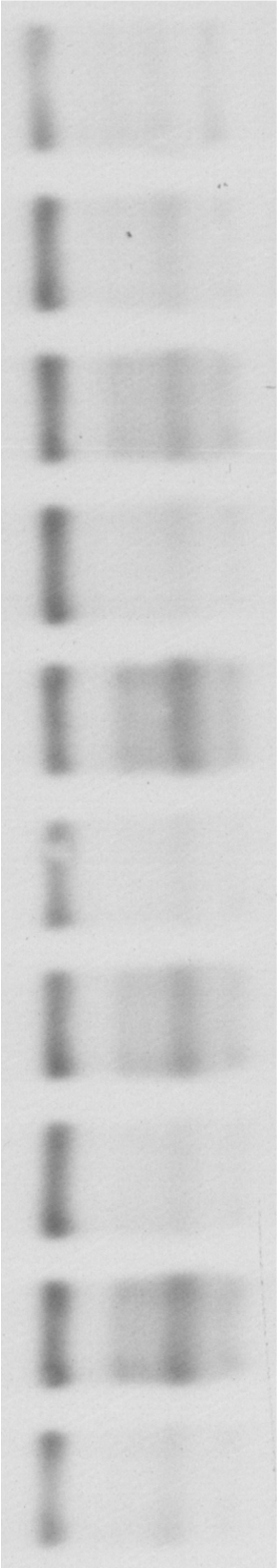

Figure 3, Panel D, western blot 5 of 5, GAPDH

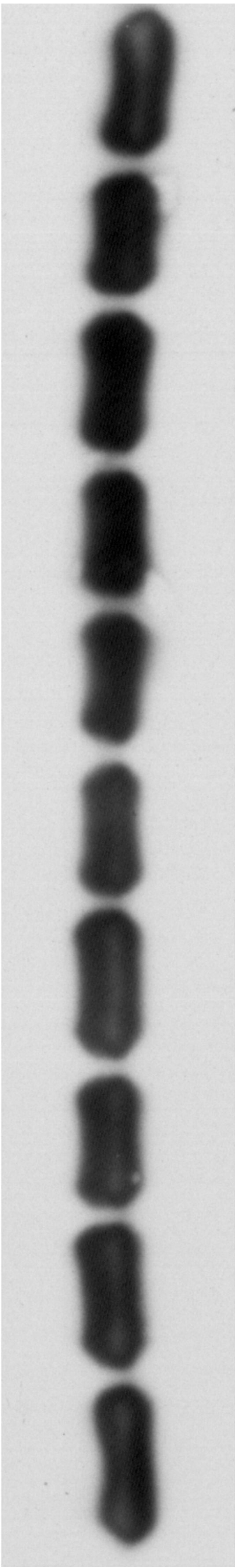

Figure 3, Panel F, western blot 1 of 2

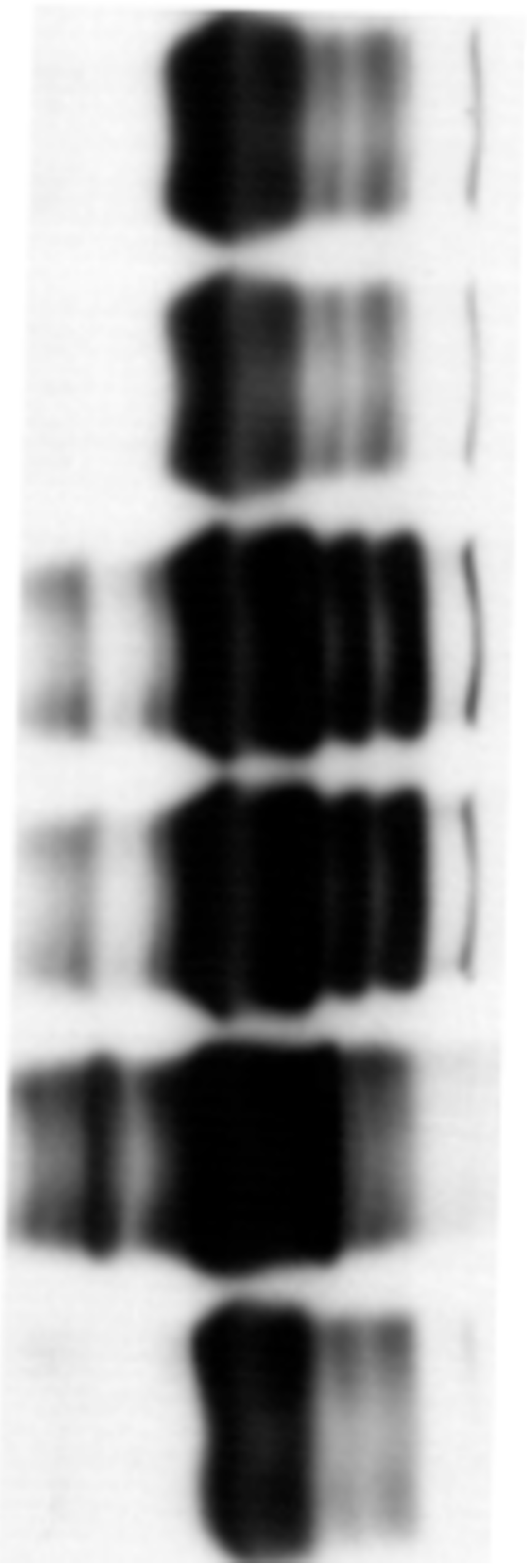

Figure 3, Panel F, western blot 2 of 2

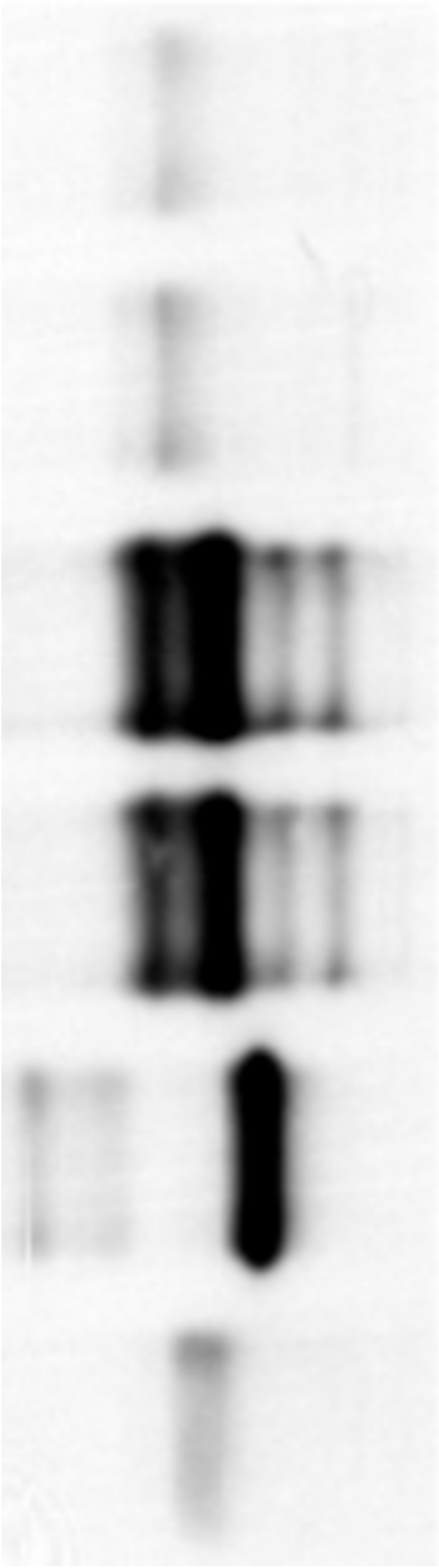

Figure 4, Panel A Coommassie-stained polyacrylamide gel

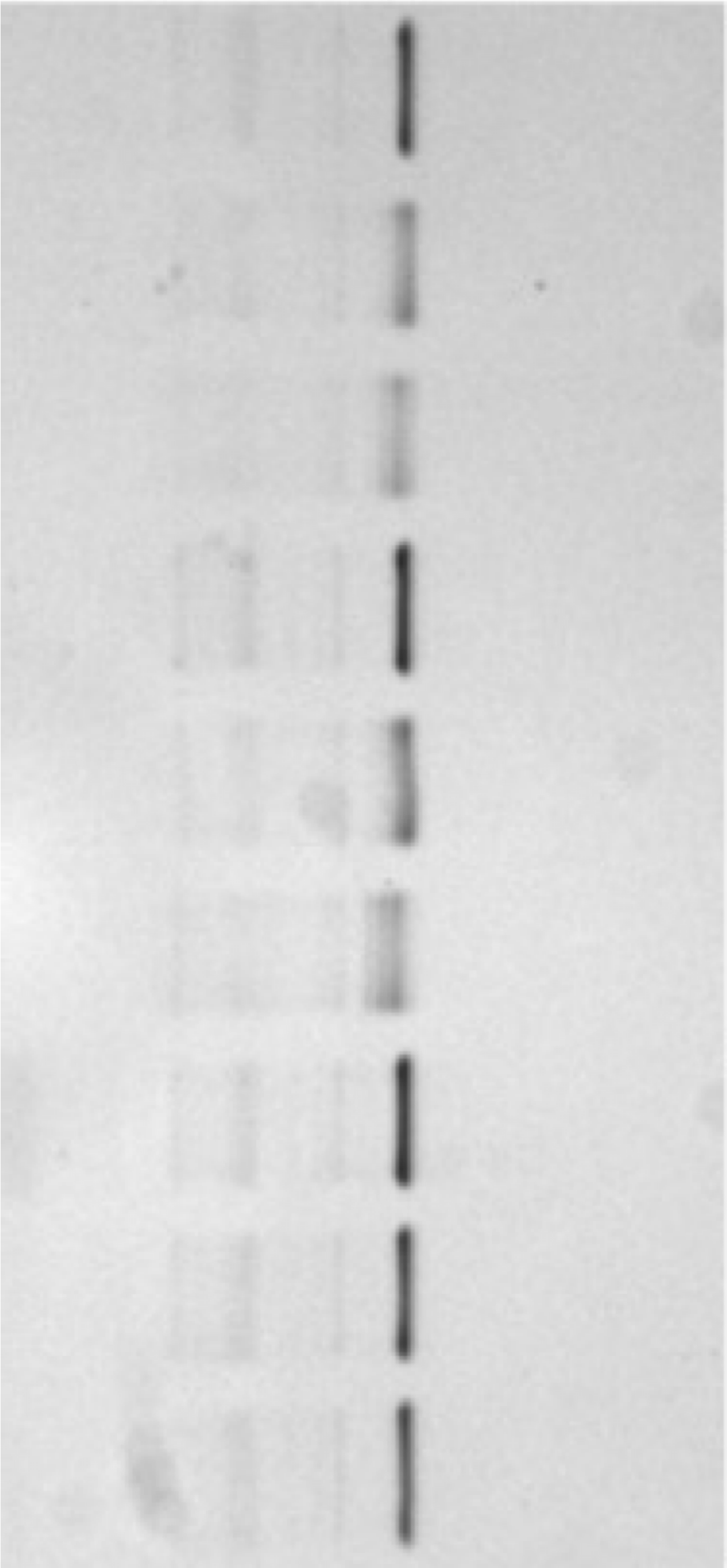

Figure 4, Panel D western blot 1 of 2, P44

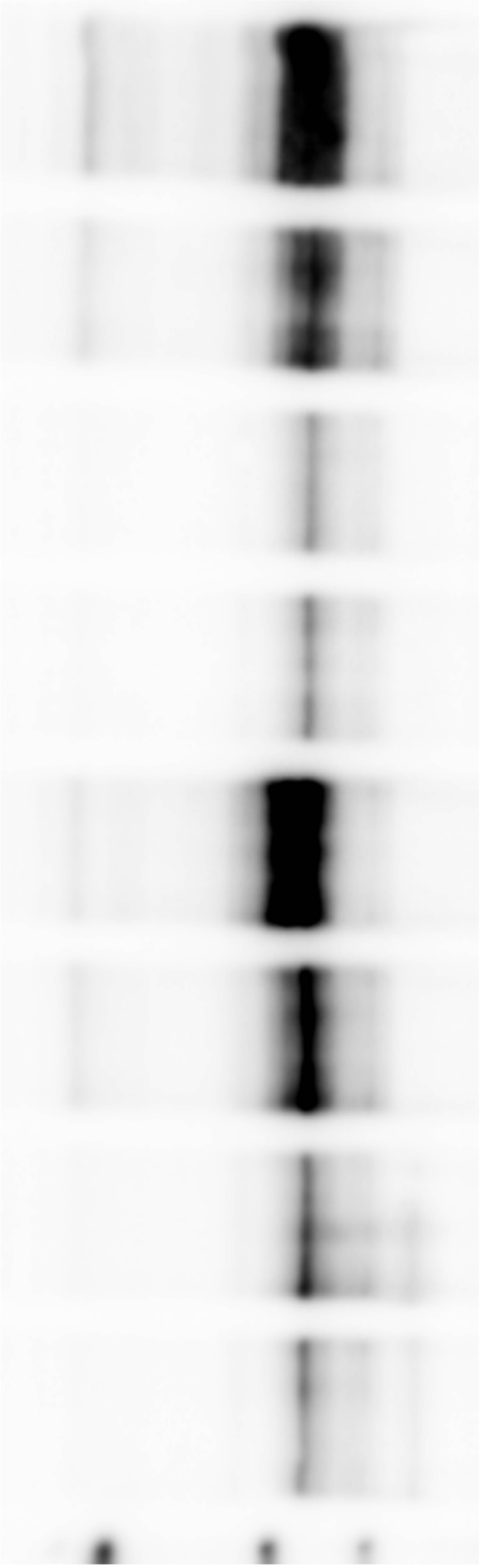

Figure 4, Panel D western blot 2 of 2, GAPDH

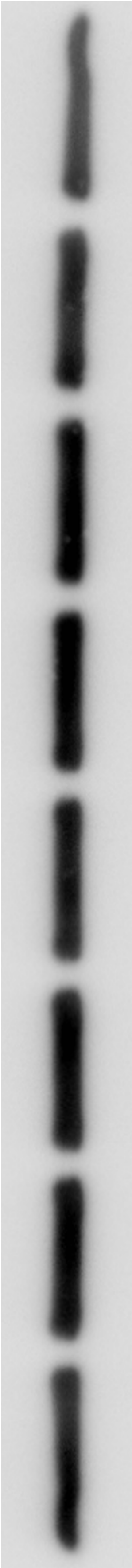

Supplement: Supplementary file 2 — Supplementary Material 2 [file 13195_2024_1443_MOESM2_ESM.pdf]
